# McKenzie treatment versus mulligan sustained natural apophyseal glides for chronic mechanical low back pain

**DOI:** 10.12669/pjms.322.9127

**Published:** 2016

**Authors:** Saira Waqqar, Syed Shakil-ur-Rehman, Shakeel Ahmad

**Affiliations:** 1 Dr. Saira Waqqar, DPT (Post Professional), Lecturer, Riphah College of Rehabilitation Sciences, Riphah International University, Islamabad, Pakistan; 2 Dr. Syed Shakil-ur-Rehman, MS (MSKPT), Principal/ Associate Professor, Riphah College of Rehabilitation Sciences, Riphah International University, Islamabad, Pakistan; 3 Dr. Shakeel Ahmad, MS (NMSKPT), Assistant Professor, Riphah College of Rehabilitation Sciences, Riphah International University, Islamabad, Pakistan

**Keywords:** McKenzie Extension Exercise Program, MulliganSustained Natural Apophyseal Glides, Chronic Mechanical Low Back Pain

## Abstract

**Background and Objective:**

Chronic mechanical low back pain is common among different age groups and genders. Different manual therapy techniques combined with exercise therapy and electrotherapy modalities play an important role in its management. Our objective was to compare the effects of McKenzie extension exercisesprogram (EEP) versus Mulligan Sustained Natural Apophyseal Glides (SNAGs) for chronic mechanical low back pain (CMLBP).

**Methods:**

This randomized control trial (RCT) was conducted at Riphah Physical Rehabilitation Centre, Pakistan Railways General Hospital Rawalpindi, from 1^st^ July to 31^st^ December 2014.

The inclusion criteria was patients of both gender and age range 30-70 years with minimum 4 weeks history of CMLBP. A total of 37 patients were screened out as per inclusion criteria and randomly placed into two groups. Twenty patients in group A were treated with Mulligan SNAGs and 17 patients in group B with McKenzie EEP for four weeks at two session per week and single session per day. Visual Analogue Scale (VAS), Oswestry Disability Scale (ODI) and lumber Range of Motion (ROM) were used as an assessment tools and were measured at baseline and at the completion 4 weeks intervention. The data was analyzed with SPSS to draw the statistical and clinical significance of both interventions.

**Results:**

At the completion of 4 weeks intervention the pre and post statistical analysis revealed that clinically the McKenzie EEP improved pain (mean 9.12 to 1.46) and disability (73.82 to 6.24) slightly more than Mulligan SNAGs (pain: from 8.85 to 2.55, disability 73.75 to 7.05), while the Mulligan SNAGs improved lumbar ROM more effectively than McKenzie EEP in all directions including flexion, extension, side bending and rotation. Statistically there was no significant difference between the effects of two interventions in managing pain and disability, and improving Lumber ROM.

**Conclusion:**

McKenzie EEP is clinically slightly more effective in the management of pain and disability as compared with Mulligan SNAGs, while Mulligan SNAGs are more effective in the improvement of lumbar ROM as compared with Mechanize EEP in the management of CMLBP.

## INTRODUCTION

Low back pain (LBP) is one of the most common ailments affecting human being. Epidemiological studies showed that 70 to 80% of all people have LBP at some time in their life.^1^ Back Pain may be mechanical or non-mechanical in nature. The mechanical conditions related with chronic low back pain consist of osteoarthritis and spinal stenosis. The non-mechanical conditions are neoplastic, infection, vascular, rheumatologist rest other multiple systemic issues.^2^

The management of low back pain consists of different intervention strategies. These include surgery, drug therapy, and non medical intervention. Most of the systematic reviews focus on the effectiveness of a single intervention and describe the effectiveness on different types of LBP.^3^

Various types of exercises have been used in the management of low back pain. For example, William’s flexion exercise, and McKenzie extension exercise are used in the treatment of low back pain.^4^ Spinal Manual Therapy (SMT) is also an effective complementary and alternative treatment for individual experiencing LBP.^5^-^7^ Spinal manual therapy is used in treatment of LBP and significant improvement in Oswestry Disability Questionnaire(ODQ) and Numeric Pain Rating Scores (NPRS)were achieved from both thrust and non thrust manipulative therapy technique, but significant differences in ODQ was obtained in favour of thrust manipulation group.^8^

One of most important technique of mobilization with movement (MWM) is SNAGs, which involve the application of accessory passive glide to lumber vertebra, while patient simultaneously perform active movement.^9^ The McKenzie extension exercise program is another method of treatment focusing on sustained posture or repeated movement, which could improve pain intensity in acute and sub acute low back pain.^10^ The current study was designed to determine the effects of Mackenzie EEP Versus Mulligan SNAGs for chronic mechanical low back pain.

## METHODS

This randomized control trial (RCT) was conducted at Riphah Physical Rehabilitation Centre, Pakistan Railways General Hospital Rawalpindi, from 1^st^ July to 31^st^ December 2014. The inclusion criteria were patients of both gender and age range 30-70 years with minimum 4 weeks history of CMLBP, while patients with acute and sub acute low back pain and trauma were excluded from the study. A total of 37 patients in which there were 20 male and 17 female patients were screened out as per inclusion criteria and randomly placed into two groups.

Twenty patients in group A were treated with Mulligan SNAGs in sitting, standing and prone position by applying anterocranial glide in the direction of treatment plane over the spinous or transverse process at 6 to 8 repetitions per sessions, and 17 patients in group B were treated with McKenzie active EEP in prone position with repeated movements along with standard protocols. Both groups were treated for four weeks at two session per week and single session per day and total 8 sessions. Treatment was applied at different lumber levels from L1 to L5. Both groups were given electrotherapy treatment such as hot pack and Transcutaneous Electrical Nerve Stimulation TENS.

The self reported Visual Analogue Scale (VAS), Oswestry Disability Scale (ODI) and lumber Range of Motion (ROM) were used as an assessment tools and were measured at baseline and at the mid intervention (2 weeks) and completion of 4 weeks intervention. The lumber ROM for flexion and extension was measured by inclinometer, side bending with finger – tip to floor method, and rotation with help of measuring tape. The data was analyzed with SPSS version 20 to draw the statistical and clinical significance of both interventions.

## RESULTS

The mean age of participants in group A was 50.25 ± 9.56 and in group B was 49.12 ± 12.47. Statistically there was no significant difference between the mean ages of participants in both the groups (P=0.762), as shown in Table-I.

**Table-I:** Participants baseline characteristics.

Demographics	Group A (Mulligan SNAGs)	Group B (McKenzie EEP)
Gender	Male=13	Male=7
	Male=7	Female=10
Mean Ages (years)	50.25±9.56	49.12±12.74

The pre and post interventional analysis revealed that clinically the patients in group B treated with McKenzie EEP improved pain slight more (mean pain from 9.12±0.48 to 1.46±1.73) as compared with the patients in group A treated with mulligan SNAGS (mean pain from 8.85±0.87 to 2.55±2.65), while statistically both the interventions were equally effective in both group for pain as assessed by VAS. as shown in Table-II.

**Table-II:** Participants Visual Analogue scale (VAS).

Study Groups	Pre VAS Mean (SD)	Post VAS Mean (SD)	Mean Difference	p-value
Group A (Mulligan SNAGs)	8.85±0.875	2.55 ± 2.65	6.20	0.000
Group B (McKenzie EEP)	9.12±.485	1.468±1.730	7.39	0.000

The pre and post interventional analysis revealed that clinically the patients in group B treated with McKenzie EEP improved function slightly more (mean ODI from 73.82±7.81 to 6.24±5.89) as compared with the patients in group A treated with mulligan SNAGS (mean ODI from 73.75±0.7.58 to 7.05±5.83), while statistically both the interventions were equally effective in both group for the management of disability as assessed by ODI.

**Table-III:** Participants Oswestry Disability Index (ODI).

Study Groups	Pre ODI Mean (SD)	Post ODI Mean (SD)	Mean Difference	p-value
Group A (Mulligan SNAGs)	73.75±7.58	7.05±5.835	66.7	0.000
Group B (McKenzie EEP)	73.82±7.812	6.24±5.890	67.58	0.000

The pre and post interventional analysis showed that clinically the patients in group A treated with Mulligan SNAGs improved lumber flexion ROM more (mean flexion from 4.00±2.902 to 56.25±3.93) as compared with the group of patients treated with McKenzie EEP (mean lumbar flexion from 4.88±3.05 to 37.94±13.35), while statistically both the interventions were equally effective in both group for the improvement of lumbar flexion as measured by inclinometer.

The pre and post interventional analysis showed that clinically the patients in group A treated with Mulligan SNAGs improved lumber extension ROM more (mean extension from 3.80±1.542 to 31.80±3.286)as compared with the group of patients treated with McKenzie EEP (mean extension 3.12±1.764 to 18.76+4.726), while statistically both the interventions were equally effective in both group for the improvement of lumbar extension as measured by inclinometer.

The pre and post interventional analysis showed that clinically the patients in group A treated with Mulligan SNAGs improved lumber side bending ROM more (mean side bending from 3.50±1.670 to 19.15±1.496) as compared with the group of patients treated with McKenzie EEP (mean side bending from 3.65±1.935 to 13.536±3.625), while statistically both the interventions were equally effective in both group for the improvement of lumbar side bending as measured by inclinometer.

**Table-IV:** Participants Lumber flexion.

Study Groups	Pre Mean (SD)	Post Mean(SD)	Mean Difference	p-value
Group A (Mulligan SNAGs)	4.00±2.902	56.25±3.932	52	0.000
Group B (McKenzie EEP)	4.88±3.059	37.94±13.35	32.25	0.000

The pre and post interventional analysis showed that clinically the patients in group A treated with Mulligan SNAGs improved lumber rotation ROM more (mean rotation from 5.00±1.5 to 16.30±2.105) as compared with the group of patients treated with McKenzie EEP (mean rotation from 3.00±1.3 to 10.41±2.12), while statistically both the interventions were equally effective in both group for the improvement of lumbar rotation as measured by inclinometer.

## DISCUSSION

The results of this study showed that both techniques were statistically effective, but clinically have slight difference in the management of pain, disability and ROM in patients with chronic low back pain. Furthermore at the completion of four weeks intervention the pre and post statistical analysis revealed that clinically the McKenzie EEP improved pain and disability slightly more than Mulligan SNAGs, while the Mulligan SNAGs improved lumbar ROM more effectively than McKenzie EEP in all directions including flexion, extension, side bending and rotation.

The result of our study showed that there is reduction in pain through McKenzie approach and long term increase in ROM through Mulligan SNAGs. In 2013 Shum GL et al. conducted a study on patient with lumber pain and decreased ROM, after the application of postero-anterior mobilization technique on lumbar spine there was immediate reduction in pain and decrease in stiffness.^11^ Our results also showed early reduction in pain and long term increase in all ROM.

Descarreaux M and colleagues in 2004 conducted a study on spinal manipulation, in which they proved that spinal manipulation is effective for the treatment of LBP. Also they suggested that care after spinal manipulation is beneficial for patient to maintain subjective post intensive treatment disability levels.^12^ In our result pre and post intervention showed no significant difference on ODI but in mid intervention showed significant difference.

**Table-V:** Participants Lumber extension.

Study Groups	Pre Lumber Extension Mean (SD)	Post lumber extension Mean(SD)	Mean Difference	p-value
Group A (Mulligan SNAGs)	3.80±1.542	31.80±3.286	27.99	0.000
Group B (McKenzie EEP)	3.12±1.764	18.76±4.726	15.64	0.000

**Table-VI:** Participants Lumber side bending.

Study Groups	Pre Lumber side bending Mean (SD)	Post Lumber side bending Mean (SD)	Mean Difference	p-value
Group A (Mulligan SNAGs)	3.50±1.670	19.15±1.496	15.65	0.000
Group B (McKenzie EEP)	3.65±1.935	13.536±3.625	9.886	0.000

**Table-VII:** Participants Lumber rotation.

Study Groups	Pre lumber rotation Mean (SD)	Post lumber rotation	Mean Difference	p-value
Group A (Mulligan SNAGs)	5.00±1.5	16.30±2.105	11.3	0.000
Group B (McKenzie EEP)	3.00±1.3	10.41±2.124	7.41	0.000

According to a recent study done by Dunning J et al. in 2015, flexion-distraction manipulation technique is an effective treatment technique for pain and disability in patient with lumber stenosis.^13^

A study conducted by Nagrale AV and colleagues has demonstrated that slump stretching and home exercises along with lumbar spine mobilization and stabilization exercises are more effective for rate and magnitude of recovery of self reported disability, pain and fear-avoidance behaviour compared to treatment without slump stretching.^14^

Another recent study in 2014 done by Mbada CE and colleagues demonstrated that health related quality of life in patients with long term mechanical low back pain decreases pain with severity. Also the addition of dynamic back extensors endurance exercises to McKenzie treatment protocol cause greater difference in health quality of life.^15^

## CONCLUSIONS

McKenzie Extension Exercise Program is clinically slightly more effective in the management of pain and disability as compared with Mulligan SNAGs, while Mulligan SNAGs are more effective in the improvement of lumbar ROM as compared with Mechanize Extension Exercise Program in the management of Chronic Mechanical Low Back Pain. Our recommendations are to conduct further studies with large sample size and long duration of intervention for the investigation of long term effects of both interventions in chronic mechanical and other types of low back pain.

## References

[1] Mbada CE, Ayanniyi O, Ogunlade SO, Orimolade EA, Oladiran AB, Ogundele AO (2014). Influence of McKenzie protocol and two modes of endurance exercises on health-related quality of life of patients with long-term mechanical low-back pain. Pan African Med J.

[2] Ahmed R, Shakil-ur-Rehman S, Sibtain F (2014). Comparison between Specific Lumber Mobilization and Core-Stability Exercises with Core-Stability Exercises Alone in Mechanical low back pain. Pak J Med Sci.

[3] Van Middelkoop M, Rubinstein SM, Kuijpers T, Verhagen AP, Ostelo R, Koes BW (2011). A systematic review on the effectiveness of physical and rehabilitation interventions for chronic non-specific low back pain. Euro Spine J.

[4] Johnson OE, Adegoke BO, Ogunlade SO (2010). Comparison of four physiotherapy regimens in the treatment of long-term mechanical low back pain. Japanese Physical Therapy Assoc.

[5] Bialosky JE, George SZ, Horn ME, Price DD, Staud R, Robinson ME (2014). Spinal manipulative therapy–specific changes in pain sensitivity in individuals with low back pain (NCT01168999). J Pain.

[6] Cuesta-Vargas A, Farasyn A, Gabel CP, Luciano JV (2014). The mechanical and inflammatory low back pain (MIL) index: development and validation. BMC Musculoskeletal Disorders.

[7] Bialosky JE, Simon CB, Bishop MD, George SZ (2012). Basis for spinal manipulative therapy: A physical therapist perspective. Electromyography Kinesiology.

[8] Kamali F, Shokri E (2012). The effect of two manipulative therapy techniques and their outcome in patients with sacroiliac joint syndrome. Bodywork Movement Therapies.

[9] Moutzouri M, Billis E, Strimpakos N, Kottika P, Oldham JA (2008). The effects of the Mulligan Sustained Natural Apophyseal Glide (SNAG) mobilisation in the lumbar flexion range of asymptomatic subjects as measured by the Zebris CMS20 3-D motion analysis system. BMC Musculoskeletal Disorders.

[10] Hosseinifar M, Akbari M, Behtash H, Amiri M, Sarrafzadeh J (2013). The Effects of Stabilization and Mackenzie Exercises on Transverse Abdominis and Multifidus Muscle Thickness, Pain, and Disability: A Randomized Controlled Trial in Nonspecific Chronic Low Back Pain. J Physical Therapy Sci.

[11] Shum GL, Tsung BY, Lee RY (2013). The immediate effect of posteroanterior mobilization on reducing back pain and the stiffness of the lumbar spine. Arch Physical Med Rehab.

[12] Descarreaux M, Blouin JS, Drolet M, Papadimitriou S, Teasdale N (2004). Efficacy of preventive spinal manipulation for chronic low-back pain and related disabilities: a preliminary study. J Manipulative Physiological Therapeutics.

[13] Dunning J, Mourad F, Giovannico G, Maselli F, Perreault T, Fernández-de-las-Peñas C (2015). Changes in Shoulder Pain and Disability after Thrust Manipulation in Subjects Presenting with Second and Third Rib Syndrome. J Manipulative Physiological Therapeutics.

[14] Nagrale AV, Patil SP, Gandhi RA, Learman K (2012). Effect of slump stretching versus lumbar mobilization with exercise in subjects with non-radicular low back pain: a randomized clinical trial. Manual Manipulative Therapy.

[15] Mbada CE, Ayanniyi O, Ogunlade SO, Orimolade EA, Oladiran AB, Ogundele AO (2014). Influence of Mc Kenzie protocol and two modes of endurance exercises on health-related quality of life of patients with long-term mechanical low-back pain. Pan African Med J.

